# Post-Exercise Recovery of Ultra-Short-Term Heart Rate Variability after Yo-Yo Intermittent Recovery Test and Repeated Sprint Ability Test

**DOI:** 10.3390/ijerph17114070

**Published:** 2020-06-07

**Authors:** Chin-Hwai Hung, Filipe Manuel Clemente, Pedro Bezerra, Yi-Wen Chiu, Chia-Hua Chien, Zachary Crowley-McHattan, Yung-Sheng Chen

**Affiliations:** 1 Physical Education Office, Fu Jen Catholic University, New Taipei City 24205, Taiwan; 088750@mail.fju.edu.tw (C.-H.H.); 055332@mail.fju.edu.tw (Y.-W.C.); 2 Escola Superior Desporto e Lazer, Instituto Politécnico de Viana do Castelo, Rua Escola Industrial e Comercial de Nun’Álvares, 4900-347 Viana do Castelo, Portugal; filipe.clemente5@gmail.com (F.M.C.); pbezerra@esdl.ipvc.pt (P.B.); 3 Instituto de Telecomunicações, Delegação da Covilhã, 1049-001 Lisboa, Portugal; 4 The Research Centre in Sports Sciences, Health Sciences and Human Development, 5001-801 Vila Real, Portugal; 5 Department of Exercise and Health Sciences, University of Taipei, Taipei 11153, Taiwan; reckosi0116@hotmail.com; 6 School of Health and Human Sciences, Southern Cross University, Lismore 2480, Australia; zac.crowley@scu.edu.au

**Keywords:** maximal intermittent exercise, post-exercise recovery, heart rate variability, autonomic nervous system

## Abstract

This study aimed to examine the agreement and acceptance of ultra-short-term heart rate (HR) variability (HRV_UST_) measures during post-exercise recovery in college football players. Twenty-five male college football players (age: 19.80 ± 1.08 years) from the first division of national university championship voluntarily participated in the study. The participants completed both a repeated sprint ability test (RSA) and a Yo-Yo intermittent recovery test level 1 (YYIR1) in a randomized order and separated by 7 days. Electrocardiographic signals (ECG) were recorded in a supine position 10 min before and 30 min after the exercise protocols. The HR and HRV data were analyzed in the time segments of baseline 5~10 min (Baseline), post-exercise 0~5 min (Post 1), post-exercise 5~10 min (Post 2), and post-exercise 25~30 min (Post 3). The natural logarithm of the standard deviation of normal-to-normal intervals (LnSDNN), root mean square of successive normal-to-normal interval differences (LnRMSSD), and LnSDNN:LnRMSSD ratio was compared in the 1st min HRV_UST_ and 5-min criterion (HRV_criterion_) of each time segment. The correlation of time-domain HRV variables to 5-min natural logarithm of low frequency power (LnLF) and high frequency power (LnHF), and LF:HF ratio were calculated. The results showed that the HRV_UST_ of LnSDNN, LnRMSSD, and LnSDNN:LnRMSSD ratio showed trivial to small effect sizes (ES) (−0.00~0.49), very large and nearly perfect interclass correlation coefficients (ICC) (0.74~0.95), and relatively small values of bias (RSA: 0.01~−0.12; YYIR1: −0.01~−0.16) to the HRV_criterion_ in both exercise protocols. In addition, the HRV_UST_ of LnLF, LnHF, and LnLF:LnHF showed trivial to small ES (−0.04~−0.54), small to large ICC (−0.02~0.68), and relatively small values of bias (RSA: −0.02~0.65; YYIR1: 0.03~−0.23) to the HRV_criterion_ in both exercise protocols. Lastly, the 1-min LnSDNN:LnRMSSD ratio was significantly correlated to the 5-min LnLF:LnHF ratio with moderate~high level (*r* = 0.43~0.72; *p* < 0.05) during 30-min post-exercise recovery. The post-exercise 1-min HRV assessment in LnSDNN, LnRMSSD, and LnSDNN:LnRMSSD ratio was acceptable and accurate in the RSA and YYIR1 tests, compared to the 5-min time segment of measurement. The moderate to high correlation coefficient of the HRV_UST_ LnSDNN:LnRMSSD ratio to the HRV_criterion_ LnLF:LnHF ratio indicated the capacity to facilitate the post-exercise shortening duration of HRV measurement after maximal anaerobic or aerobic shuttle running. Using ultra-short-term record of LnSDNN:LnRMSSD ratio as a surrogate for standard measure of LnLF:LnHF ratio after short-term bouts of maximal intensity field-based shuttle running is warranted.

## 1. Introduction

Heart rate (HR) variability (HRV) is a physiological process that reflects the biological fluctuation in cardiac activation that is regulated by the autonomic nervous system (ANS). Heart rate variability assessment requires biosignal recording via non-invasive techniques to detect beat-to-beat intervals of the HR responses in a time series. Patterns of HRV have previously been used to measure the cardiovascular function in response to exercise adaptation [1,2], psychological performance [3], environmental behavior [4], and recovery status in sports training [5,6]. 

To validate HRV recordings, the Task Force of the European Society of Cardiology and the North American Society of Pacing Electrophysiology recommend a series of 512 R-wave to R-wave intervals (RRI) for HRV data analysis [7]. Alternatively, short-term 5-min HRV recordings after a 5-min stabilization (maintain a fixed posture in a stable manner) can be used as a standard process during HRV measurements [7]. The standardization of testing procedure requires around 10 min to obtain sufficient number of RRI for time-domain, frequency-domain, and non-liner analyses. However, this process is very time-consuming and is mainly limited to clinical application; therefore, this type of analysis is seldom used in applied sports settings. Thus, there is a need for more time-efficient methods for analyzing HRV in applied sports contexts.

Post-exercise recovery of cardiac-related responses play a critical role in the homeostatic functioning of the ANS and cardiovascular system. The capacity to recover from exercise-induced ANS changes is highly related to the exercise intensity [8,9]. Repeated sprint ability tests (RSA), which are anaerobic-based assessments of exercise capacity, may induce post-exercise effects on vagal withdrawal. For example, Abad et al. [10] found that the depression of parasympathetic reactivation may be longer than 2-h during passive recovery after the RSA test. In contrast, aerobic assessments, such as the Yo-Yo intermittent recovery level 1 test (YYIR1), have shown no significant influence on post-exercise modulation of cardiac autonomic function [1]. To understand the potential difference in post-exercise recovery of HRV between anaerobic and aerobic exercise, Nakamura et al.’s study [11] compared the HRV recovery between the 30–15 intermittent fitness test (a similar test of aerobic function to the YYIR1) and the RSA test in 13 national male handball players during the preparatory period of National Championship. The relative change in HRV recovery after 5-min stabilization between the 30–15 intermittent fitness test and RSA test was similarly altered, with fast HRV recovery of the root mean square of successive normal-to-normal interval differences (RMSSD) after 3 min of the RSA test. Due to these contradictory findings, further research is therefore needed to elucidate the differences in HRV between aerobic and anaerobic assessment and training.

To improve the utility of HRV measures in the assessment of exercise recovery in applied sports settings, ultra-short-term HRV (HRV_UST_) assessment has become attractive to practitioner and researches due to the time efficiency of data collection. HRV_UST_ only requires 10–60 s recording times, depending upon the methodologies and device technology. It is recognized that 1-min HRV_UST_ displays excellent validity, reliability, and limits of agreements as a surrogate to the 5-min criterion of HRV record (HRV_criterion_) [12,13,14,15,16,17]. The advantage of HRV_UST_ record has been demonstrated in sports training [16,17], exercise testing [12,13,18], cardiovascular medicine [19,20,21], and metabolic disease [22].

In frequency-domain HRV analysis, the ratio of low-frequency power (LF), and high-frequency power (HF) (LF:HF ratio) is recognized as an essential HRV parameter to indicate the sympathovagal responses [7]. However, most studies examining the validity and accuracy of HRV_UST_ demonstrate inconsistencies in LF, HF, and LF:HF ratio within 60-s time segments, compared to HRV_criterion_ [19,23,24,25]. In contrast, in time-domain HRV analysis, excellent agreement and accuracy of HRV_UST_ has been demonstrated in the standard deviation of normal-to-normal intervals (SDNN) and RMSSD [19,23,24,25]. Esco and his colleagues [26] reported that 1-min HRV_UST_ of the natural logarithm of SDNN:RMSSD (LnSDNN:LnRMSSD) ratio was significantly correlated (0.72~0.86, very large correlation) to 5-min HRV_criterion_ of natural logarithm of the LF:HF (LnLF:LnHF) ratio at both rest and recovery following a maximal graded exercise test in an athletic population. Following their finding, a potential implementation to use the HRV_UST_ of SDNN:RMSSD ratio as a surrogate to integrate the sympathovagal responses after maximal intensity exercise is feasible. Therefore, it is necessary to compare and contrast these two methods in relation to post-exercise evaluation of various exercise modalities.

The physical tests, such as RSA and YYIR1, are extensively used to evaluate physical capacities and training adaptations in team sports [27,28]. Considering the lack of information regarding post-exercise HRV_UST_ records after field-based exercises, the primary purpose of this study was to investigate the agreement and acceptance of post-exercise time-domain (LnSDNN and LnRMSSD) and frequency-domain (LnLF and LnHF) HRV_UST_ measures after field-based shuttle running assessments (the RSA and YYIR1) in colleague male football players. The secondary purpose was to determine the relationship of ultra-short-term and short-term recordings of LnSDNN:LnRMSSD ratio to LnLF:LnHF ratio during post-exercise recovery. Based on findings in the previous studies, it was hypothesized that time-domain HRV_UST_ would show better agreement and reproducibility to the 5-min criterion of HRV record (HRV_criterion_) than that of frequency-domain HRV. It was also hypothesized that HRV_UST_ of LnSDNN:LnRMSSD ratio would demonstrate significant correlations to HRV_criterion_ of LnLF:LnHF ratio during post-exercise recovery in both exercise protocols.

## 2. Materials and Methods

### 2.1. Experimental Approach to the Problem

A pretest-posttest crossover design was used to examine the post-exercise HRV_UST_ after the RSA and YYIR1 exercise protocols. The period of HRV assessment was divided into baseline, post-exercise 0~5 min (Post 1), post-exercise 5~10 min (Post 2), and post-exercise 25~30 min (Post 3). The time segments of HRV recordings consisted of the first 60-s (HRV_UST_) and the 5-min criterion (HRV_criterion_). The nLnSDNN, LnRMSSD, and LnSDNN:LnRMSSD ratio were used in time-domain analysis. In addition, the LnLF,LnHF, and LnLF:LnHF ratio were used in frequency-domain analysis. The relationship of LnSDNN:LnRMSSD ratio and LnLF:LnHF ratio was examined at baseline and post-exerciser time points. Since the RSA and YYIR1 tests are common tools for exercise testing and fitness training, assessment of post-exercise HRV can help to understand the autonomic function during recovery. Comparison of these two methods could help us to improve our current knowledge of post-exercise recovery strategy.

**Figure 1:**
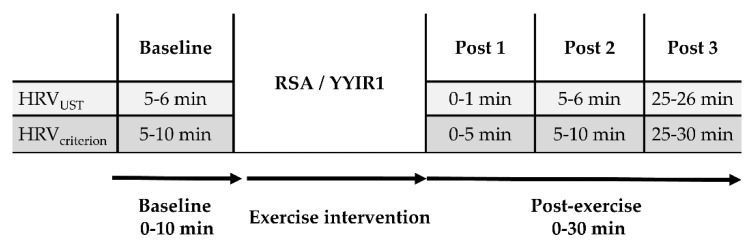
Schematic presentation of experimental procedure in the present study. RSA = repeated sprint ability test; YYIR1 = Yo-Yo intermittent recovery test level 1; HRV_UST_ = ultra-short-term heart rate variability; HRV_criterion_ = criterion of heart rate variability.

The first 5-min ECG data in the baseline were discarded to prevent orthostatic effect as a standard process of HRV record. Figure 1 presents the experimental procedures in the present study.

### 2.2. Experimental Procedure

Participants first visited the exercise performance laboratory for a familiarization. The first visit consisted of an experimental introduction, procedural habituation, and determination of physical characteristics. The second and third visits were experimental visits for the RSA and YYIR1 exercise protocols with 3~7 days apart. The sequence order of the exercise protocols was conducted via an online randomizer website (https://www.randomizer.org/). The participants were asked not to undertake vigorous exercise 24 h before the visits and to avoid caffeine-containing substances and smoking 2 h before the experiments.

Initially, the participants were prepared with a lead II ECG. The skin of the participants were cleaned with alcohol wipes prior to electrode application (Kendall™ 200 Series Foam Electrodes; Covidien, Mansfield, MA, USA). During the baseline measurement (10-min ECG recording), the participants were required to lie supine on a medical bed in a quiet research room. Subsequently, the participants performed a 5-min warm-up cycling exercise, which consisted of 50-watt power output with a pedaling rate of 60 revolutions per minute (Optibike Med; Ergoline, Germany). Afterwards, the participants wore their personal sports shoes and performed the RSA and YYIR1 exercise protocols indoors on artificial surface. After the exercise termination, post-exercise ECG was recorded immediately in a supine position for 30 min. Room temperature was controlled at 25 °C, and humidity was set within the range of 50~60%.

### 2.3. Participants

The sample size estimation was determined based on a priori type of power analysis using G*Power 3.1.9.4 software (G*Power, Düsseldorf, Germany ) [29]. A means differences between two dependent measures with power of 80% and an alpha value of 0.05 in the two-tailed test were set to estimate the minimum number of participants. Based on a previous study [26], it indicated that a minimum of 10 participants was required to approach actual power of 0.85.

Twenty-five male college outfield football players voluntarily participated in the study (mean ± standard deviation: age = 19.80 ± 1.08 years; height = 173.87 ± 5.60 cm; body weight = 67.91 ± 8.17 kg; body fat = 16.04 ± 5.05%; players experience = 10.20 ± 1.66 years). The inclusion criteria included: (1) undertake regular football training at least five times a week; (2) a minimum football training experience of five years; and (3) currently playing in the first division of a national university championship. Exclusion criteria included: (1) any history of severe neuromuscular injury; (2) lower extremity injury within six months; and (3) current neurological or cardiovascular diseases. All players signed informed consent forms and were familiarized with experimental procedures one week before the experiment proper. The study has been approved by the Institute Review Board of the hospital (2014-06-003CC) and was conducted in accordance with the Declaration of Helsinki with its later amendments.

### 2.4. Heart Rate Variability

ECG lead set (SS2LB, Biopac Inc., Goleta, CA, USA) with a conventional lead II arrangement was used to record cardiac responses in a supine position. A data acquisition system (MP35, Biopac Inc., Goleta, CA, USA) was used to collect resting ECG signals for 10 min before warm up and 30 min immediately after the RSA and YYIR1 exercise protocols. The analog signals of ECG were transformed into digital signals by using an analog-to-digital converter with a sampling rate of 1000 Hz via the Biopac Student Lab system. The ECG waveforms were then filtered using Kubios HRV analysis software Premium version 3.2.0. (Kubios, Kuopio, Finland) to calculate the time-domain and frequent-domain HRV indices. The artefact correction of RRI was set at a threshold of medium level, and window width was set at 300 s, with window overlap of 50%. Smoothing priors set at 500 Lambda were used for the detrending method [30]. The area-under-the-curve of the spectral peaks within the range of 0.04~0.15 Hz and 0.15~0.40 Hz were set for LF and HF, respectively. The power spectra of RR intervals were calculated by means of Fast Fourier Transformation. 

### 2.5. Yo-Yo Intermittent Recovery Test Level 1

The YYIR1 test consisted of 20-m shuttle runs back and forth between two lines, with a gradual incremental increase in speed over time. A jogging distance of 5 m recovery zone was set behind the start line, with participants given 10-s resting recovery after each bout of shuttle running. The running speed was controlled by digital audio bleeps from a laptop. The running speed of the first four bouts (0-m~160-m) was set at 10~13 km^.^h^−1^, and the subsequent seven bouts (160-m~440-m) were 13.5–14 km^.^h^−1^. After the 11th bout, the running speed increased by increments of 0.5 km^.^h^−1^ every eight bouts (i.e., after 760-m, 1080-m, 1400-m, 1720-m, etc.). The total covered distance was recorded when the participants failed to return to the start line a second time (a verbal warning was given to the participants in the first instance that they failed to make the line in time).

The validity of the YYIR1 test to assess aerobic capacity has previously been described [28] with test-retest reliability between 0.78 to 0.98 [31]. Each participant’s predicted maximal oxygen consumption was calculated by the equation: distance in meters × 0.0084 + 36.4 [28].

### 2.6. Repeated Sprint Ability Test

The RSA test consisted of a distance of 20-m sprint back and forth, repeated 6 times with 20-s rest intervals between bouts. The participants were allowed to conducted passive recovery or jogging behind the starting line during the 20-s rest interval. Timing gates (Fusion Sport, Brisbane, Queensland, Australia) were placed 30-cm behind the starting line and set at a height of 1.2-m. Two preliminary trials to familiarize participants with the RSA protocol was given followed by a 5 min rest.

Sprint time for each RSA performance was recorded. The best and worst sprints times were recorded as RSA_best_ and RSA_worst_, respectively. The mean sprint time of RSA test (RSA_mean_) was calculated as the average of the six sprints times. The mean of the first, second, and third sprint times were recorded as the RSA_1–3mean_, while the mean of the fourth, fifth, and sixth sprints were recorded as the RSA_4–6mean_. The percent decrement of RSA was calculated by using the equation: 100 – (RSA_totoal_/(RSA_best_ × 6) × 100). Fatigue index was calculated by using the formulae: (RSA_worst_ − RSA_best_)/RSA_best_ × 100 [32].

The validity and reliability of the RSA test to assess the 20-m shuttle sprint ability in football players has previously been reported with an interclass correlation coefficients (ICC) value of 0.81 for RSA_mean_ [33].

### 2.7. Statistical Analyses

Descriptive data of the measured variables are presented as mean and standard deviation (SD). The normality of all variables of interest were assessed via Kolmogorov–Smirnov statistical tests. HRV variables are commonly found to be non-normal distributions. To adjust for this violation, a natural logarithm was used for HRV comparisons. Inter-differences of HRV_UST_ to criterion was analyzed by using effect size (ES) calculations. The level of ES was interpreted as trivial (0.0~0.2), small (0.2~0.6), moderate (0.6~1.2), large (1.2~2.0), and very large (>2.0) [34]. For reliability analysis, ICC with two-way random model and single measures were used to determine relative values of reliability. The level of ICC values were expressed as nearly perfect (0.9~1), very large (0.70~89), large (0.50~69), moderate (0.31~49), and small (0~0.3) [34]. Furthermore, the Bland–Altman plots were used to evaluate the upper and lower limits of agreements between the HRV_UST_ and the HRV_criterion_ among time segments. In addition, the relationship of LnSDNN:LnRMSSD ratio and LnLF:LnHF ratio between HRV_UST_ and HRV_criterion_ during baseline, Post 1, Post 2, and Post 3 in both exercise protocols were assessed by using Pearson product-moment correlation coefficient (*r*). The threshold level of the correlation coefficient was determined as trivial (*r* < 0.1), small (0.1 < *r* < 0.3), moderate (0.3 < *r* < 0.5), high (0.5 < *r* < 0.7), very high (0.7 < *r* < 0.9), nearly perfect (*r* > 0.9), and perfect (*r* = 1) [34]. Statistical analyses were conducted using by SPSS^®^ Statistics version 25.0 (IBM, Armonk, NY, USA) and Microsoft Excel 2016 (Microsoft Corporation, Redmond, WA, USA).

## 3. Results

### 3.1. Exercise Performance

Table 1 shows the descriptive statistics regarding exercise performance during the RSA and YYIR1 exercise protocols.

### 3.2. Heart Rate Variability

The results of the LnSDNN, LnRMSSD, and LnSDNN:LnRMSSD ratio during baseline, Post 1, Post 2, and Post 3 in the RSA and YYIR1 exercise protocols are presented in Table 2. The result of HRV_UST_ and HRV_criterion_ comparison demonstrated trivial and small ES (LnSDNN = RSA: −0.06 trivial~−0.23 small, YYIR1: 0.10 trivial~−0.32 small; LnRMSSD = RSA: −0.07 trivial~−0.16 trivial, YYIR1: −0.17 trivial~0.26 small; and LnSDNN:LnRMSSD ratio = RSA: −0.00 trivial~0.18 trivial, YYIR1: −0.15 trivial~0.49 small). The ICC values showed very large and nearly perfect correlation in all comparisons, except LnSDNN:LnRMSSD ratio in the RSA baseline [0.69 (0.36; 0.83) large] and YYIR1 Post 3 [0.63 (0.27; 0.83) large]. In addition, limits of agreements showed relatively small values of bias in all comparisons [RSA: 0.01 (−0.07; 0.08)~−0.12 (−0.66; 0.41); YYIR1: −0.01 (−0.06; 0.05)~−0.16 (−0.57; 0.26)].

The result of HRV_UST_ and HRV_criterion_ comparison demonstrated trivial and small ES (LnLF = RSA: −0.13 trivial~−0.54 small, YYIR1: 0.18 trivial~−0.47 small; LnHF = RSA: 0.07 trivial~0.52 small, YYIR1: −0.06 trivial~0.33 small; and LnLF:LnHF ratio = RSA: −0.12 trivial~−0.44 small, YYIR1: −0.04 trivial~−0.38 small) (Table 3). The ICC values showed large variations in the RSA test (−0.02 small~0.64 large) and YYIR1 test (0.22 small~0.68 large). In terms of the Bland–Altman analysis, the result showed limits of agreements showed relatively small bias in all comparisons [RSA: −0.02 (−0.60; 0.55)~0.65 (−4.19; 5.53); YYIR1: 0.03 (−0.92; 0.98)~−0.23 (−1.15; 0.67)].

**Figure 2:**
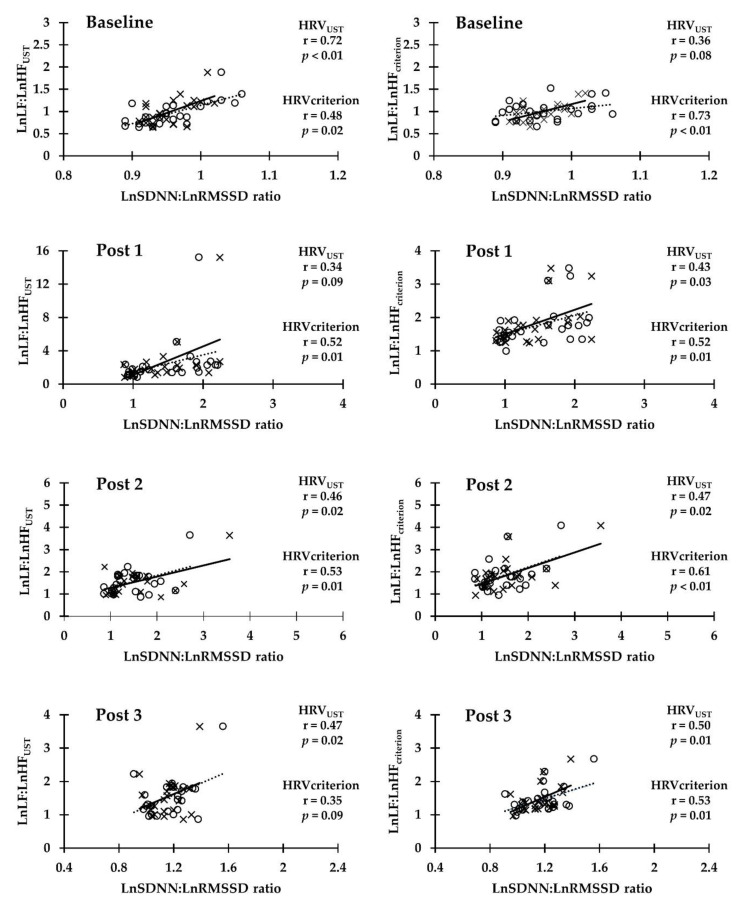
Pearson correlation coefficient between LnSDNN:LnRMSSD ratio and LnLF:LnHF ratio (1 min and criterion) at baseline, post exercise 0–5 min (Post 1), post exercise 5–10 min (Post 2), and post exercise 25–30 min (Post 3) in repeated sprint ability test. Scatter plots between 1 min LnSDNN:LnRMSSD ratio and LnLF:LnHF ratio are presented as open circles (dotted line). Scatter plots between criterion of LnSDNN:LnRMSSD ratio and LnLF:LnHF ratio are presented as cross marks (solid line).

**Figure 3:**
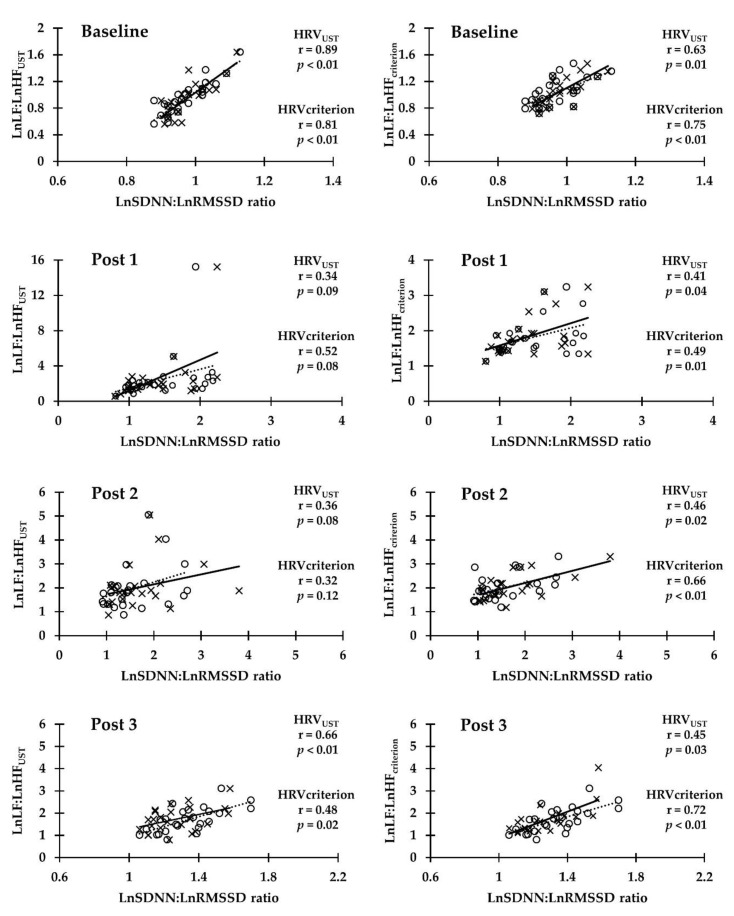
Pearson correlation coefficient of the relationship between LnSDNN:LnRMSSD ratio and LnLF:LnHF ratio (1 min and criterion) at baseline, post exercise 0–5 min (Post 1), post exercise 5–10 min (Post 2), and post exercise 25–30 min (Post 3) in Yo-Yo intermittent recovery test level 1. Scatter plots between 1 min LnSDNN:LnRMSSD ratio and LnLF:LnHF ratio are presented as open circles (dotted line). Scatter plots between criterion of LnSDNN:LnRMSSD ratio and LnLF:LnHF ratio are presented as cross marks (solid line).

The results showed a broad range of Pearson correlation coefficient between the HRV_UST_ and HRV_criterion_ of LnSDNN:LnRMSSD ratio and the HRV_UST_ and HRV_criterion_ LnLF:LnHF ratio at different time points in either the RSA test (*r* = 0.34 moderate, *p* = 0.09~*r* = 0.73 very high, *p* < 0.01) or the YYIR1 test (*r* = 0.32 moderate, *p* = 0.12~*r* = 0.89 very high, *p* < 0.01). The HRV_UST_ LnSDNN:LnRMSSD ratio and the HRV_criterion_ LnLF:LnHF ratio was significantly highly correlated with RSA Post 3 and YYIR1 Baseline. Moderate significant correlations were found between RSA Post 1 and 2, and YYIR1 Post 1, 2, and 3. In addition, the HRV_criterion_ comparison between the LnSDNN:LnRMSSD ratio and LnLF:LnHF ratio was significantly very highly correlated with RSA Baseline, YYIR1 Baseline, and YYIR1 Post 3. High significant correlations were found between RSA Post 1, 2, and 3, and YYIR1 Post 1. A moderate significant correlation was found at YYIR1 Post 2 (Figure 2 and Figure 3).

## 4. Discussion

The primary purpose of this study was to examine the agreement and acceptance of post-exercise HRV_UST_ parameters in LnSDNN, LnRMSSD, LnLF, and LnHF after field-based RSA and YYIR1 exercises. The secondary purpose was to determine the correlation of ultra-short-term and short-term measures of the LnSDNN:LnRMSSD ratio to the LnLF:LnHF ratio during 30-min post-exercise recovery. The main findings of this study included: (1) 1-min HRV_UST_ in time-domain variables (LnSDNN, LnRMSSD, and LnSDNN:LnRMSSD ratio) showed excellent validity and reliability to the standard 5-min HRV assessment after the short-term bouts of anaerobic-based and aerobic-based intermittent running exercises. (2) A large variation of ICC values and correlation outputs in frequency-domain HRV variables (i.e., LnLF, LnHF, and LnLF:LnHF ratio) indicated an inconsistence of post-exercise HRV assessment in ultra-short-term and short-term evaluation. (3) One-minute and 5-min LnSDNN:LnRMSSD ratio scores were moderately to highly correlated with the 5-min LnLF:LnHF ratio scores during passive post-exercise recovery after maximal intensity short-term shuttle running.

### 4.1. Time-Domain Analysis

The findings in the present study supported our hypothesis that time-domain HRV_UST_ would demonstrate excellent acceptance and reproducibility to the HRV_criterion_. The accuracy and agreement of 1-min LnSDNN, LnRMSSD, and LnSDNN:LnRMSSD to the traditional 5-min HRV_criterion_ measure was observed despite maximal exercise intensity after the RSA and YYIR1 tests. Moreover, our data revealed a gradual enhancement of the LnSDNN, LnRMSSD, and LnSDNN:LnRMSSD ratio when recovery time progressed after the RSA and YYIR1 tests. The augmentation of these time-domain variables indicates an increase in parasympathetic reactivation and a decrease in sympathetic activity during recovery [35]. Specifically, a rebound of LnSDNN, LnRMSSD, and LnSDNN:RMSSD ratio were observed at Post 3 in both exercise protocols. Previously, feasibility and agreement of 1-min HRV_UST_ to the standard 5-min HRV record was based on observations during resting measurement [12,13,15,16,17,36]. However, current knowledge regarding the methodology of the post-exercise HRV_UST_ is not well understood and has not been extensively explored. Esco et al. [12] investigated the reliability and limits of agreement of 10-s, 30-s, and 1-min HRV_UST_ during resting and after a maximal graded exercise test (Bruce protocol) in 23 college athletes. The results showed that 10-s and 30-s shorter time segments of HRV records decrease the agreement and hence the validity of the measurement compared to the standard 5-min HRV_criterion_. However, acceptable levels of validity, and tight limits of agreement, were found in the 1-min HRV_UST_ during resting and post-exercise recovery. Another recent study revealed high reproducibility of test-retest 30-s HRV_UST_ recordings via LnSDNN, LnRMSSD parameters at the beginning of passive or active recovery after maximal graded cycling test [37]. As demonstrated in our findings, the HRV_UST_ measures showed trivial and small ES, very large and nearly perfect ICC values, and a very high level of correlation to HRV_criterion_, even at the beginning of the post-exercise recovery. This observation may support the time efficient use of HRV assessment after maximal intensity of field-based shuttle running exercise. Since the SDNN and RMSSD are vagal-related HRV indices, the application of HRV_UST_ assessment to evaluated post-exercise parasympathetic reactivation may be considered in future research [35].

### 4.2. Frequency-Domain Analysis

It is interesting to note that the HRV_UST_ of LnLF, LnHF, and LnLF:LnHF showed trivial to small ES, small to large ICC, and trivial to very high correlation when compared to the HRV_criterion_ in both exercise protocols. Our observations were in agreement with previous studies showing inaccurate measures of LF, HF, and LF:HF ratio in less than 60 s [19,23,24,25]. It appears that the large variation and inconsistent outcomes could limit the implementation of frequency-domain analysis in post-exercise assessment. One of the possible explanations to our finding is that the LF, HF, and LF:HF ratio is thought of as a sensitive measure to the respiratory frequency and thus could potentially undermine its validity and accuracy in HRV_UST_ measurement in our study [38]. In addition, the underlying mechanisms to determine post-exercise HRV modulation include sympathetic withdraw, parasympathetic reactivation, metaboreflex stimulation, baroreflex activity, and vascular regulation [35]. These mechanical factors may profoundly affect the frequency-domain variables in our study. It is also important to note that the length of data acquisition can influence the frequency-domain analysis [7]. Collectively, our findings suggest not to use the ultra-short-term records of LF, HF, and LF:HF ratio to evaluate HRV recovery.

### 4.3. Correlation Coefficient

The HRV_UST_ of LnSDNN:LnRMSSD ratio demonstrates significant correlations with HRV_criterion_ of LnLF:LnHF ratio during post-exercise recovery in both exercise protocols. The 1-min LnSDNN:LnRMSSD ratio was significantly correlated to 5-min LnLF:LnHF ratio with moderate~high level during 30-min post-exercise recovery. The correlation coefficient between the HRV_UST_ of LnSDNN:LnRMSSD ratio and the HRV_criterion_ of LnLF:LnHF ratio found in the present study led us to accept our secondary hypothesis. The advantage in using time-domain HRV indices is that it allows for high reproducibility when compared to that of frequency-domain HRV indices during post-exercise recovery [37]. The ratio of SDNN:RMSSD as an alternative of LF:HF ratio has previously been recommended in a longitudinal observation [39] and a cross-sectional study [26]. Esco and colleagues [26] reported that 1-min HRV_UST_ of the LnSDNN:LnRMSSD ratio was significantly correlated (0.72~0.86) to 5-min HRV_criterion_ of LnLF:LnHF ratio at rest and recovery following a maximal graded exercise test in athletic population. Although the level of correlation was lower than those observed in Esco et al.’s study, an alternative of using LnSDNN:LnRMSSD ratio to frequency computations for evaluating post-exercise recovery in relation to sympathovagal balance is warranted.

### 4.4. Limitation

The limitations of this study were that: (1) the time window between study participation and training periodization varied among the participants. The fitness level and psychological status among the participants could be a potentials bias in this study. (2) Transition between the exercise termination and post-exercise HRV measurement was required in our experimental setting. The transition period may lead to a time delay of the true measurement at Post 1 point. (3) A homogeneous group of college male football players participated in this study. Application to general population and different sports athletes might be beyond the scope of the present study.

### 4.5. Practical Implications

The functional implication of the present study included that HRV_UST_ measures were acceptable to use immediately after shot-term shuttle running when the exercise intensity approached maximal intensity. The HRV is recognized as a convenient tool to assess the cardiac-related heath and recovery capacities in sports sciences and medicine. Implementation of 1-min post-exercise HRV assessment could help the profession of strength and conditioning to manage recovery duration when multiple rounds of maximal intensity exercise bouts are used.

Furthermore, strength and conditioning practitioners attempt to manage the efficiency of physical testing schedule in elite sports training. In fact, maximal intensity of aerobic or anaerobic tests are usually conducted in a separate day (i.e., RSA and YYIR1) in order to avoid potential fatigue to affect outcome of measurement. Monitoring post-exercise recovery via HRV_UST_ measures might be an alternative to understand the optimal time allocation among testing protocols (resting interval between the tests). Future studies are needed to investigate the relationship of HRV recovery and aerobic-based and anaerobic-based performance associated with rest duration.

## 5. Conclusions

In conclusion, for accuracy of HRV measurements, the post-exercise 1-min HRV assessment in LnSDNN, LnRMSSD, and LnSDNN:LnRMSSD ratio was a valid and reliable alternative to the 5-min HRV in either the RSA test or the YYIR1 test. Using the 1-min HRV assessment as a surrogate to the 5-min HRV should be cautioned in the RSA and the YYIR1 exercise protocols due to a wide range of ICC values during resting states and post-exercise recovery. The moderate to high correlation coefficient of 1-min and 5-min LnSDNN:LnRMSSD ratios to the 5-min LnLF:LnHF ratios indicate a potential ability to utilize the shortened HRV measurements after short-term anaerobic or aerobic shuttle running.

## Figures and Tables

**Table 1:** Descriptive data of exercise performance of the participants.

Exercise Performance	Mean ± SD	CV (%)
RSA_1–3__mean_ (s)	7.31 ± 0.29	3.91
RSA_4–6__mean_ (s)	7.64 ± 0.27	3.47
RSA_mean_ (s)	7.48 ± 0.26	3.46
RSA_total_ (s)	44.86 ± 1.55	3.46
RSA_best_ (s)	7.17 ± 0.28	3.85
RSA_worse_ (s)	7.75 ± 0.26	3.30
RSA_decrement_ (%)	4.26 ± 1.94	45.55
RSA fatigue index (%)	8.13 ± 3.38	41.53
YYIR1 distance (m)	1214.40 ± 290.95	23.96
YYIR1 VO2_max_ (ml^.^kg^−1.^min^−1^)	46.60 ± 2.44	5.25

SD = Standard deviation; CV = coefficient of variation; RSA = repeated sprint ability; YYIR1 = Yo-Yo intermittent recovery test level 1; s = seconds; m = meters; VO2_max_ = maximal oxygen consumption; ml^.^kg^−1.^min^−1^ = milliliter per kilogram per minute.

**Table 2:** Natural logarithm of standard deviation of normal-to-normal intervals (LnSDNN), root mean square differences between adjacent normal R-R intervals (LnRMSSD), and LnSDNN:LnRMSSD ratio during 0–1 min and 0–5 min of time segments of the baseline, post exercise 0–5 min, 5–10 min, and 25–30 min in the repeated sprint ability test and YoY o intermittent recovery test level 1 protocols.

Period	Parameter	HRV_UST_ (Mean ± SD)	HRV_criterion_ (Mean ± SD)	ES (95% CI)	ICC (95% CI)	Bias (±1.96 SD)
RSA Baseline	LnSDNN	3.89 ± 0.34	3.91 ± 0.37	−0.06 (0.61; 0.50) *	0.82 (0.64; 0.92) ‡	−0.02 (−0.43; 0.40)
	LnRMSSD	4.06 ± 0.43	4.09 ± 0.42	−0.07 (0.63; 0.48) *	0.90 (0.78; 0.95) §	−0.03 (−0.41; 0.36)
	LnSDNN:LnRMSSD	0.96 ± 0.05	0.96 ± 0.04	−0.00 (−0.55; 0.55) *	0.69 (0.36; 0.83) †	0.01 (−0.07; 0.08)
RSA Post 1	LnSDNN	1.62 ± 0.39	1.69 ± 0.44	−0.17 (−0.72; 0.39) *	0.88 (0.74; 0.94) ‡	−0.07 (−0.46; 0.33)
	LnRMSSD	1.27 ± 0.64	1.36 ± 0.61	−0.14 (−0.70; 0.42) *	0.88 (0.76; 0.95) ‡	−0.08 (−0.66; 0.49)
	LnSDNN:LnRMSSD	1.47 ± 0.48	1.39 ± 0.42	0.18 (−0.38; 0.73) *	0.81 (0.60; 0.91) ‡	0.08 (−0.47; 0.63)
RSA Post 2	LnSDNN	1.69 ± 0.52	1.82 ± 0.52	−0.23 (−0.78; 0.33) #	0.84 (0.65; 0.93) ‡	−0.12 (−0.66; 0.41)
	LnRMSSD	1.34 ± 0.66	1.40 ± 0.68	−0.09 (−0.64; 0.47) *	0.83 (0.65; 0.92) ‡	0.06 (−0.73; 0.84)
	LnSDNN:LnRMSSD	1.45 ± 0.48	1.51 ± 0.61	−0.11 (−0.66; 0.45) *	0.82 (0.60; 0.91) ‡	−0.07 (−0.74; 0.61)
RSA Post 3	LnSDNN	2.81 ± 0.53	2.89 ± 0.51	−0.15 (−0.71; 0.40) *	0.95 (0.85; 0.98) §	−0.09 (−0.39; 0.21)
	LnRMSSD	2.45 ± 0.65	2.55 ± 0.60	−0.16 (−0.71; 0.40) *	0.95 (0.87; 0.98) §	−0.10 (−0.44; 0.25)
	LnSDNN:LnRMSSD	1.17 ± 0.15	1.15 ± 0.11	0.15 (−0.40; 0.71) *	0.90 (0.71; 0.94) §	0.02 (−0.12; 0.16)
YYIR1 Baseline	LnSDNN	3.93 ± 0.34	3.88 ± 0.29	0.16 (−0.40; 0.71) *	0.90 (0.78; 0.95) §	0.05 (−0.22; 0.32)
	LnRMSSD	4.05 ± 0.40	3.98 ± 0.41	0.17 (−0.38; 0.73) *	0.93 (0.82; 0.97) §	0.07 (−0.21; 0.35)
	LnSDNN:LnRMSSD	0.97 ± 0.07	0.98 ± 0.06	−0.15 (−0.71; 0.40) *	0.89 (0.78; 0.95) ‡	−0.01 (−0.06; 0.05)
YYIR1 Post 1	LnSDNN	1.48 ± 0.32	1.53 ± 0.33	−0.15 (−0.71; 0.40) *	0.84 (0.68; 0.93) ‡	−0.05 (−0.41; 0.30)
	LnRMSSD	1.11 ± 0.44	1.23 ± 0.47	−0.26 (−0.82; 0.30) #	0.84 (0.64; 0.93) ‡	−0.12 (−0.59; 0.36)
	LnSDNN:LnRMSSD	1.47 ± 0.45	1.38 ± 0.42	0.20 (−0.35; 0.76) *	0.80 (0.59; 0.91) ‡	0.09 (−0.44; 0.62)
YYIR1 Post 2	LnSDNN	1.42 ± 0.45	1.56 ± 0.40	−0.32 (−0.89; 0.23) #	0.76 (0.48; 0.89) ‡	−0.14 (−0.68; 0.40)
	LnRMSSD	1.03 ± 0.46	1.09 ± 0.47	−0.13 (−0.68; 0.43) *	0.82 (0.64; 0.92) ‡	−0.06 (−0.61; 0.49)
	LnSDNN:LnRMSSD	1.56 ± 0.56	1.65 ± 0.68	−0.14 (−0.70; 0.41) *	0.74 (0.50; 0.88) ‡	−0.09 (−0.96; 0.78)
YYIR1 Post 3	LnSDNN	2.62 ± 0.52	2.67 ± 0.50	−0.10 (−0.65; 0.46) *	0.92 (082; 0.96) §	−0.05 (−0.45; 0.35)
	LnRMSSD	2.01 ± 0.57	2.16 ± 0.59	−0.26 (−0.81; 0.30) #	0.90 (0.66; 0.96) §	−0.16 (−0.57; 0.26)
	LnSDNN:LnRMSSD	1.34 ± 0.17	1.26 ± 0.15	0.49 (−0.07; 1.06) #	0.63 (0.27; 0.83) †	0.07 (−0.17; 0.32)

The level of effect size was symbolled as trivial (0.0–0.2) as *, small (0.2–0.6) as #, moderate (0.6–1.2) as = †, large (1.2–2.0) as ‡, very large (>2.0) as §. The level of interclass correlation coefficients was denoted small (0–0.3) as *, moderate (0.31–49) as #, large (0.50–69) as †, very large (0.70–89) as ‡, and nearly perfect (0.9–1) as §. RSA = repeated sprint ability test; YYIR1 = Yo-Yo intermittent recovery test level 1; HRV_UST_ = ultra-short-term heart rate variability; HR_criterion_ = criterion of heart rate variability; SD = standard deviation; CI = confident interval; ES = effect size; ICC = intraclass correlation coefficient.

**Table 3:** Natural logarithm of low frequency power (LnLF), high frequency power (LnHF), and LnLF:LnHF ratio during 0–5 min of time segments of the baseline, post exercise 0–5 min, 5–10 min, and 25–30 min in the repeated sprint ability test and Yo-Yo intermittent recovery test level 1 protocols.

Period	Parameter	HRV_UST_ (Mean ± SD)	HRV_criterion_ (Mean ± SD)	ES (95% CI)	ICC (95% CI)	Bias (±1.96 SD)
RSA Baseline	LnLF	3.70 ± 0.51	3.81 ± 0.40	−0.24 (−0.80; 0.32) #	0.27 (−0.13; 0.59) *	−0.11 (−1.21; 0.98)
	LnHF	3.90 ± 0.50	3.87 ± 0.40	0.07 (−0.49; 0.62) *	0.36 (−0.04; 0.66) #	−0.03 (−0.99; 1.04)
	LnLF:LnHF	0.98 ± 0.29	1.01 ± 0.22	−0.12 (−0.67; 0.44) *	0.36 (−0.05; 0.66) #	−0.02 (−0.60; 0.55)
RSA Post 1	LnLF	4.35 ± 0.28	4.39 ± 0.15	−0.18 (−0.73; 0.38) *	0.39 (0.01; 0.68) #	−0.04 (−0.53; 0.44)
	LnHF	2.56 ± 0.99	2.70 ± 0.65	−0.17 (−0.72; 0.39) *	0.57 (0.24; 0.78) †	−0.14 (−1.67; 1.38)
	LnLF:LnHF	2.44 ± 2.81	1.77 ± 0.63	0.32 (−0.23; 0.89) #	0.25 (−0.13; 0.58) *	0.65 (−4.19; 5.53)
RSA Post 2	LnLF	4.26 ± 0.28	4.39 ± 0.18	−0.54 (−1.12; 0.02) #	−0.02 (−0.35; 0.35) *	−0.13 (−0.78; 0.52)
	LnHF	3.04 ± 0.74	2.66 ± 0.69	0.52 (−0.04; 1.09) #	0.32 (−0.04; 0.62) #	−0.38 (−1.97; 1.22)
	LnLF:LnHF	1.53 ± 0.58	1.82 ± 0.71	−0.44 (−1.01; 0.12) #	0.56 (0.21; 0.78) †	−0.29 (−1.42; 0.85)
RSA Post 3	LnLF	4.27 ± 0.27	4.30 ± 0.16	−0.13 (−0.69; 0.42) *	0.44 (0.06; 0.71) #	−0.03 (−0.61; 0.55)
	LnHF	3.02 ± 0.73	3.11 ± 0.56	−0.14 (−0.69; 0.42) *	0.58 (0.25; 0.79) †	−0.09 (−1.36; 1.18)
	LnLF:LnHF	1.54 ± 0.58	1.45 ± 0.39	−0.18 (−0.37; 0.74) *	0.64 (0.34; 0.82) †	0.09 (−0.73; 0.91)
YYIR1 Baseline	LnLF	3.64 ± 0.57	3.87 ± 0.37	−0.47 (−1.04; 0.09) #	0.48 (0.12; 0.73) #	−0.23 (−1.15; 0.67)
	LnHF	3.95 ± 0.44	3.81 ± 0.40	0.33 (−0.23; 0.89) #	0.61 (0.30; 0.81) †	0.13 (−0.58; 0.85)
	LnLF:LnHF	0.95 ± 0.26	1.04 ± 0.21	−0.38 (−0.94; 0.18) #	0.58 (0.25; 0.79) †	−0.09 (−0.50; 0.33)
YYIR1 Post 1	LnLF	4.34 ± 0.44	4.43 ± 0.10	−0.27 (−0.84; 0.28) #	0.28 (−0.11; 0.60) *	−0.09 (−0.84; 0.66)
	LnHF	2.46 ± 0.95	2.61 ± 0.57	−0.19 (−0.75; 0.37) *	0.61 (0.30; 0.81) †	−0.14 (−1.49; 1.21)
	LnLF:LnHF	2.49 ± 2.80	1.81 ± 0.55	0.33 (−0.22; 0.89) #	0.26 (−0.13; 0.58) *	0.68 (−4.11; 5.47)
YYIR1 Post 2	LnLF	4.40 ± 0.21	4.47 ± 0.08	−0.43 (−1.00; 0.12) #	0.22 (−0.15; 0.54) *	−0.08 (−0.47; 0.32)
	LnHF	2.55 ± 0.79	2.35 ± 0.53	0.29 (−0.26; 0.85) #	0.47 (0.12; 0.72) #	0.20 (−1.15; 1.55)
	LnLF:LnHF	1.98 ± 0.94	2.01 ± 0.53	−0.04 (−0.60; 0.51) *	0.42 (0.03; 0.70) #	−0.04 (−1.66; 1.58)
YYIR1 Post 3	LnLF	4.33 ± 0.28	4.37 ± 0.14	−0.18 (−0.74; 0.38) *	0.43 (0.05; 0.70) #	−0.05 (−0.51; 0.42)
	LnHF	2.79 ± 0.77	2.83 ± 0.64	−0.06 (−0.61; 0.50) *	0.60 (0.27; 0.80) †	−0.04 (−1.30; 1.22)
	LnLF:LnHF	1.70 ± 0.57	1.67 ± 0.62	0.05 (−0.50; 0.61) *	0.68 (0.39; 0.85) †	0.03 (−0.92; 0.98)

The level of effect size was symbolled as trivial (0.0–0.2) as *, small (0.2–0.6) as #, moderate (0.6–1.2) as = †, large (1.2–2.0) as ‡, very large (>2.0) as §. The level of interclass correlation coefficients was denoted small (0–0.3) as *, moderate (0.31–49) as #, large (0.50–69) as †, very large (0.70–89) as ‡, and nearly perfect (0.9–1) as §. RSA = repeated sprint ability test; YYIR1 = Yo-Yo intermittent recovery test level 1; HRV_UST_ = ultra-short-term heart rate variability; HRV_criterion_ = criterion of heart rate variability; SD = standard deviation; CI = confident interval; ES = effect size; ICC = intraclass correlation coefficient.
